# Electrostatic interplay: The interaction triangle of polyamines, silicic acid, and phosphate studied through turbidity measurements, silicomolybdic acid test, and ^29^Si NMR spectroscopy

**DOI:** 10.3762/bjnano.5.211

**Published:** 2014-11-06

**Authors:** Anne Jantschke, Katrin Spinde, Eike Brunner

**Affiliations:** 1 TU Dresden, Fachrichtung Chemie und Lebensmittelchemie, Bioanalytische Chemie, 01062 Dresden, Germany

**Keywords:** phosphate, self-assembly, silica–polyamine interactions, silicomolybdic acid test, ^29^Si NMR, turbidity measurements

## Abstract

The discovery of long-chain polyamines as biomolecules that are tightly associated to biosilica in diatom cell walls has inspired numerous in vitro studies aiming to characterize polyamine–silica interactions. The determination of these interactions at the molecular level is of fundamental interest on one hand for the understanding of cell wall biogenesis in diatoms and on the other hand for designing bioinspired materials synthesis approaches. The present contribution deals with the influence of amines and polyamines upon the initial self-assembly processes taking place during polyamine-mediated silica formation in solution. The influence of phosphate upon these processes is studied. For this purpose, sodium metasilicate solutions containing additives such as polyallylamine, allylamine and others in the presence/absence of phosphate were investigated. The analyses are based mainly on turbidity measurements yielding information about the early aggregation steps which finally give rise to the formation and precipitation of silica.

## Introduction

Long-chain polyamines (LCPAs) were previously found biomolecules that are tightly associated to the biosilica of various diatom species [1–5]. They consist of linear oligo-propyleneimine chains attached to putrescine or spermine [5–6]. Biosilica-associated LCPAs occur either as free molecules [1,4] or covalently attached to the ε-amino groups of certain lysine-residues [7–8] in highly post-translationally modified peptides, so-called silaffins [7–10]. It is, furthermore, remarkable that the amine moieties in LCPAs from diatoms are partially methylated. The degree of methylation depends on the diatom species. LCPAs have also been identified in the silica spicules of sponges [11] and thus appear to be a general component for biological silica formation. In vitro experiments with LCPAs extracted from diatom biosilica revealed that these molecules are capable of enhancing the silica precipitation from silicic acid solutions [2,5]. It is very remarkable that the silica precipitation process is extremely rapid if the solutions contain phosphate or other suitable counterions in addition to LCPAs and silicic acid [2–3,12]. These observations have inspired numerous in vitro investigations to understand the underlying self-assembly processes and interactions [13–30]. Corresponding in vitro investigations using polyallylamine (in form of polyallylamine hydrochloride, PAH) as a synthetic analogue [15] for native LCPAs revealed that phosphate is capable of inducing the self-assembly of PAH into large aggregates that could be detected by dynamic light scattering (DLS) experiments [16–17]. Self-assembled PAH aggregates were shown to strongly enhance the speed of silica precipitation, which takes place at a time scale of seconds or minutes. In the absence of phosphate, solutions containing polyallylamine and silicic acid are capable of forming so-called polyamine-stabilized silica sols [18]. These stabilized sols exhibit particles of 30–50 nm diameter, which remain stable up to 24 h. In the relevant pH range of 5–7, monosilicic acid is an uncharged molecule, Si(OH)_4_ [31]. However, monosilicic acid (p*K*_a_ ≈ 9.8) spontaneously transforms into higher oligomers and silica particles (p*K*_a_ ≈ 6–7) [19] which exhibit a negative surface charge in solution. It was, therefore, suggested that the stabilized sol consists of polyamine–silica nanoparticle superstructures resulting from a self-assembly process driven by attractive interactions between positively charged polyamines and negatively charged silica particles [18,20]. Subsequent in vitro studies support the idea of polyamine-stabilized sols [21–22]. In contrast to the charged PAH, uncharged polymers such as polyvinylpyrrolidone or polyethylene glycol cannot undergo such a self-assembly process driven by electrostatic interactions [23–24]. However, they interact with the silicic acid/silica species via hydrogen bonding and possibly hydrophobic interactions. These interactions even result in the stabilization of mono- and disilicic acid species [22–23]. The described observations have meanwhile lead to numerous biomimetic or bioinspired silica synthesis approaches [21,25–32].

The addition of negatively charged phosphate ions (see above) to pure LCPA solutions has already been studied in detail. Phosphate results in a cross-linking of the positively charged LCPAs. The resulting self-assembly processes give rise to the formation of a microemulsion finally leading to macroscopic phase separation. It was concluded that the phosphate-driven self-assembly processes are accelerating the silica-precipitation processes. However, the self-assembly processes going on in LCPA/silicic acid/phosphate solutions have not yet been studied in detail — in contrast to the pure LCPA/phosphate system [12,16–17]. Further understanding of the molecular interactions between polyamines, silicic acid/silica species and phosphate is, therefore, a rewarding research topic. The aim of the present study is the analysis of the influence of the polyamine structure and charge upon the polycondensation of silicic acid in the absence and presence of phosphate. The kinetics of the aggregation and silica polycondensation processes were studied by a combination of turbidity measurements and silicomolybdic acid test [31,33–34]. The study includes the monomeric allylamine, its fully methylated analogue allyltrimethylammonium bromide (allylamineQ) and the widely used long-chain model polyamine poly(allylamine) hydrochloride (PAH). Moreover, a homologous series of diamines with different degree of methylation was studied in order to visualize the possible influence of hydrophobic interactions. For ^29^Si NMR spectroscopy aqueous solutions of isotope-labelled sodium [^29^Si] metasilicate as precursor compound were used. Different silica precursors, such as toxic TMOS (tetramethyl orthosilicate) or TEOS (tetraethyl orthosilicate), have been used for previous in vitro experiments. Here we used the biorelevant sodium metasilicate as silicic acid precursor. Sodium metasilicate dissolves in water to silicic acid (Na_2_SiO_3_ + 3H_2_O → Si(OH)_4_ + NaOH) at a pH value of 11.5–12.5 and can subsequently be acidified. Another benefit of using sodium metasilicate is the relatively high sodium concentration since it is known that silicon uptake and transport are connected with the sodium metabolism of diatoms [35–36].

## Results and Discussion

Two of the most important parameters influencing the polycondensation reaction of silicic acid [31] are concentration [37] and pH value [38]. The maximum polycondensation rates occur around pH 7 [31,38]. It should be noted that the formation of diatom cell walls takes place in the so-called silica deposition vesicle (SDV) with an internal pH of 5–6 [39–42]. Previous in vitro experiments were carried out by Sumper et al. at pH 6.8 [3,27]. Other experimentalists have chosen pH 5.5 [2,23–24]. We have therefore carried out experiments at both pH values, ca. 7 and 5.5.

**Figure 1:**
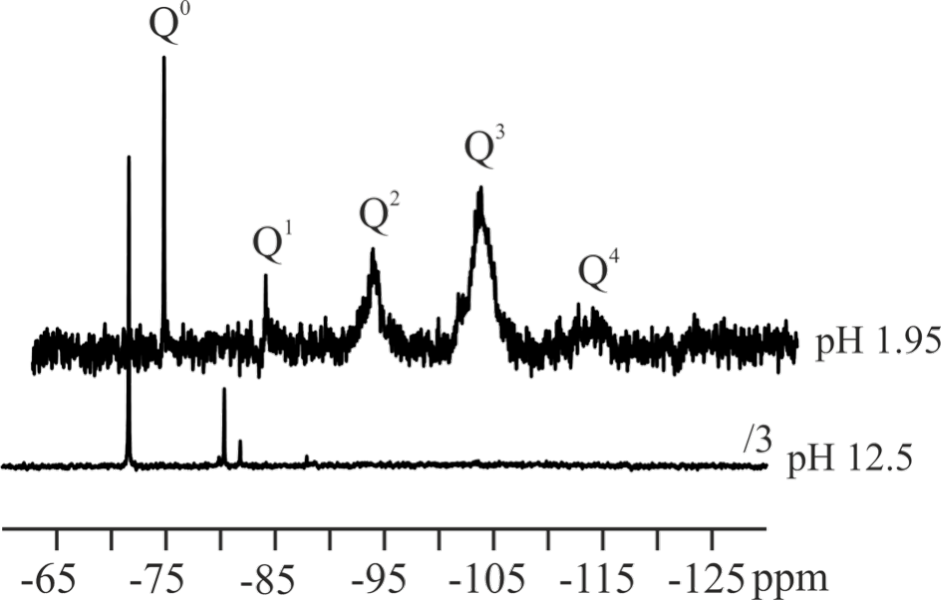
^29^Si HR NMR spectra of the control stock solution (75 mM SiO_2_) in acidic and basic solution.

At pH > 12, the silicic acid solutions (in the form of silicate) remain stable even at high concentrations [23,31]. The same is true under very acidic conditions. However, ^29^Si HR NMR measurements (Figure 1) of sodium metasilicate solutions without any additive reveal that the state of the silicic acid is different for the basic (pH 12.5) and the acidic environment (pH 1.95). In the basic environment, the signals of Q_0_ and Q_1_ are dominating the spectrum (Q*_n_* = Si(OSi)*_n_*(OH)_4−_*_n_*, *n* = 0–4) [43]. Note that highly mobile species exhibit narrow signals as observed in basic solution. In contrast, the spectrum of the acidic solution already exhibits the Q_2_, Q_3_, and Q_4_ signals characteristic for higher oligomers which are broadened due to an increasing degree of immobilization. That means the condensation reaction is more advanced in the acidic solution whereas the basic solution mainly consists of Q_0_ and Q_1_ species. It should be noted that these two species are rapidly interconverting. The sum of Q_0_ and Q_1_ represents the so-called “soluble silica” and can be detected by the silicomolybdic acid test reaction [31,33–34]. In the basic solution, practically all silica is soluble, i.e., molybdate-reactive. For this reason, our experiments were carried out starting from a basic sodium metasilicate solution.

**Figure 2:**
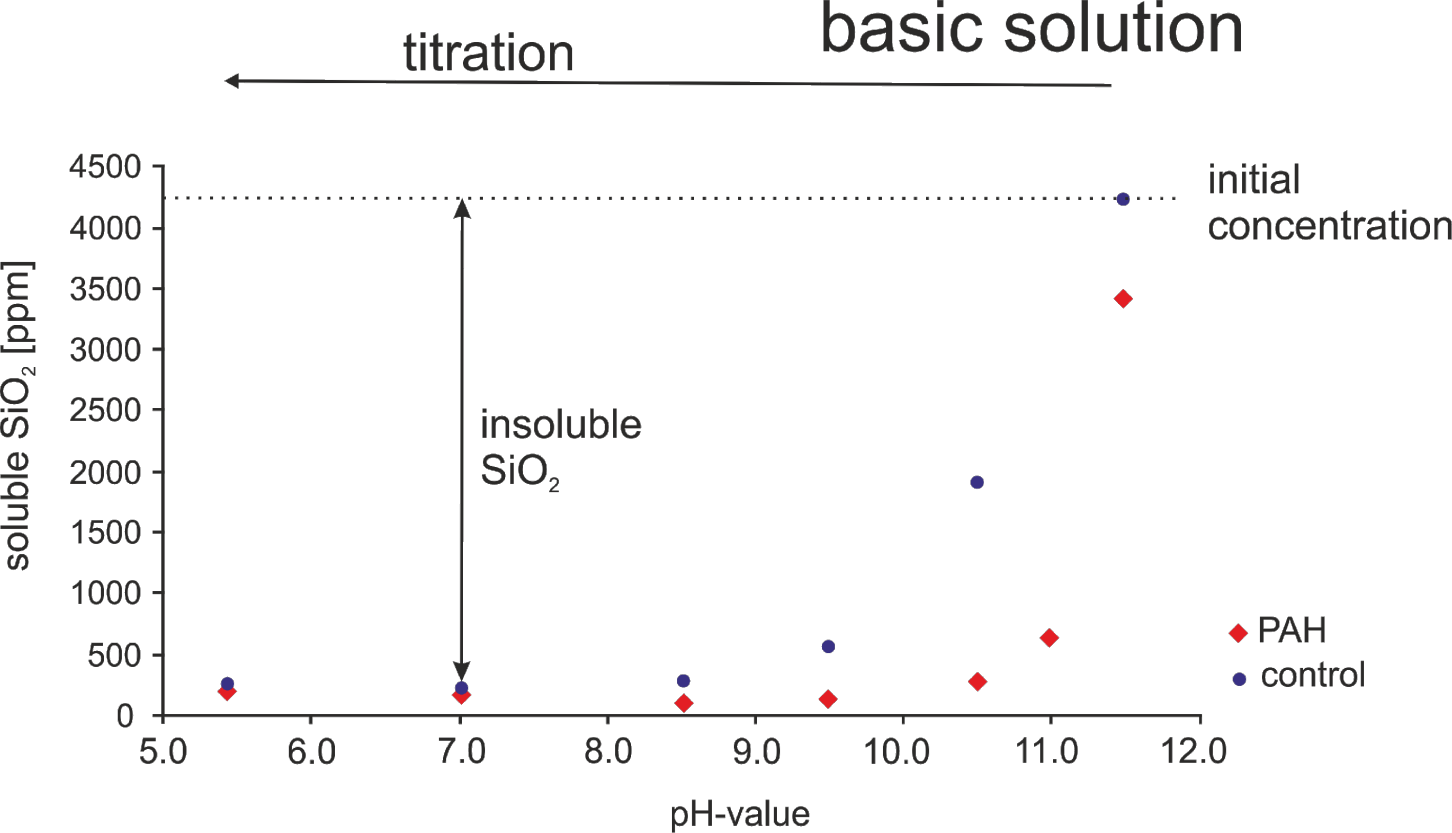
Soluble silicic acid concentration at different pH values in the presence (red rhombuses) and absence (control, blue circles) of PAH. SiO_2_ concentration 70 mM (4200 ppm); PAH concencation 0.45 mM. The silicomolybdic acid test reaction was performed 30 min after sample preparation/pH titration.

Figure 2 shows the concentration of soluble silica as a function of pH determined by the silicomolybdic acid test. A pure sodium metasilicate solution and a solution containing sodium metasilicate and PAH are compared. The interaction between silicic acid/silica species and PAH is assumed to be mediated by the NH_2_ groups. Therefore, the PAH concentration was chosen to obtain a Si/N ratio of 1:1. In the pure sodium metasilicate solution, all silica is detected by the silicomolybdic acid test reaction at pH 11.5. However, the amount of soluble silica steadily decreases with decreasing pH. In the pH range of 5–7, more than 95% of the initial silicic acid are present in the form of insoluble silica. This is the result of the silica polycondensation reaction, which transforms soluble silica species into insoluble silica species, i.e., higher oligomers or silica nanoparticles. Such insoluble silica species are not detected by the silicomolybdic acid test. It is remarkable that the presence of PAH strongly influences the amount of soluble silica, i.e., the silica polycondensation reaction. The concentration of soluble silica in the PAH-containing sample is always lower than in the PAH-free control. The most pronounced difference between the pure and the PAH-containing sodium metasilicate solution occurs in the pH range between 11.5 and 8.5. The p*K*_a_ of PAH amounts to ca. 9.7 [44–45] and the p*K*_a_ of Si(OH)_4_ to circa 9.8. That means PAH is positively charged for pH < 9.7 and Si(OH)_4_ is negatively charged for pH > 9.8. Hence, purely electrostatic interactions between the polyamine and monosilicic acid cannot be expected.

In contrast to monosilicic acid, higher silicic acid oligomers/silica nanoparticles exhibit lower p*K*_a_ values. For fumed silica, two different types of silanol groups are reported with p*K*_a_ values of ca. 8 and 4.5 [46–47]. It can therefore be assumed that, in the relevant pH range of 5–7, the silica nanoparticles exhibit a negative surface charge. Electrostatic interactions will, therefore, occur between the positively charged polyamine and negatively charged higher silicic acid oligomers/silica nanoparticles below the p*K*_a_ of PAH. The resulting immobilization of higher silicic acid oligomers could indeed be observed by ^29^Si NMR spectroscopy previously [23]. The soluble silica (mono- and disilicic acid) is almost completely polycondensed into insoluble species (higher silica oligomers/nanoparticles) below pH 9 after 30 min in pure sodium metasilicate as well as the PAH containing sample (see Figure 2). The PAH-containing sample exhibits a white precipitate whereas the pure sodium metasilicate solution has formed a gel.

### Turbidity measurements

**Figure 3:**
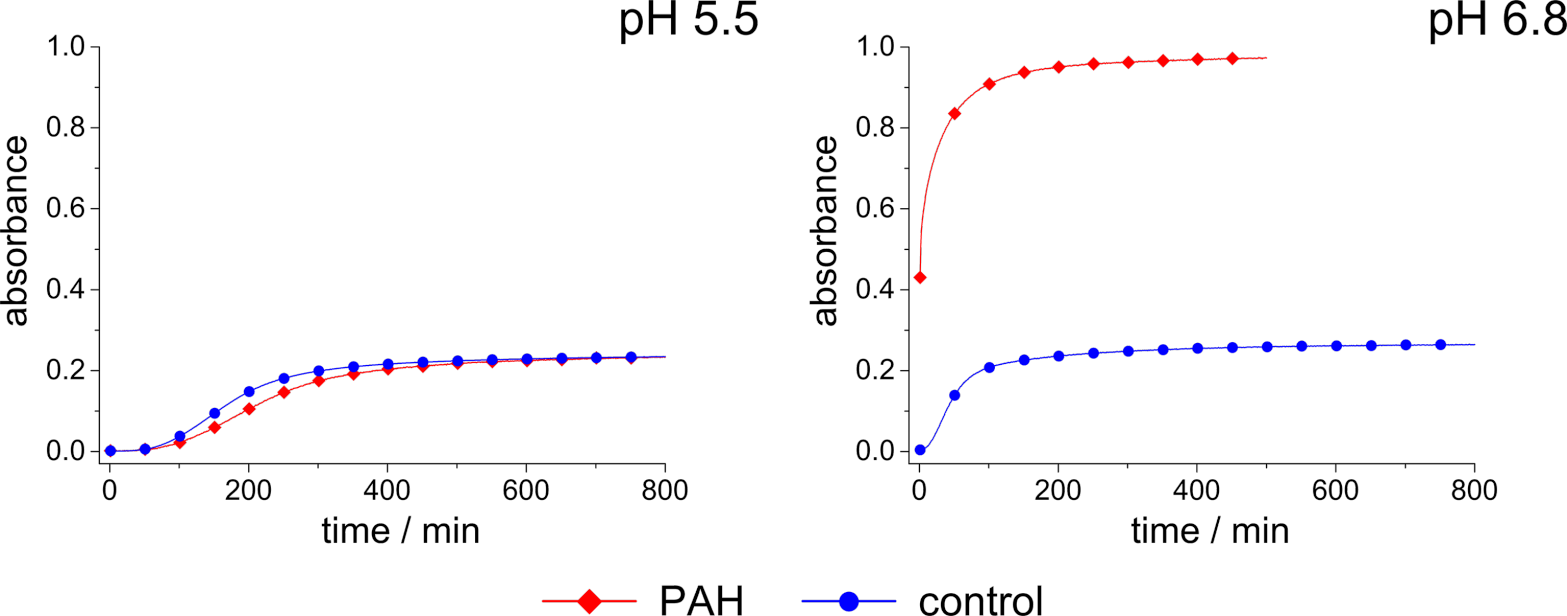
Absorbance of a 90 mM sodium metasilicate solution with 31.25 µM PAH (red rhombuses) and of a pure sodium metasilicate solution (control, blue circles) measured as a function of time up to 800 min.

Turbidity measurements provide a simple possibility for the time-resolved study of self-assembly processes in solutions containing organic molecules and silicic acid as has been demonstrated by Robinson et al. [48]. The process of self-assembly and silica polycondensation reaction increases the turbidity of the solution, which causes an increasing absorption. This property can easily be determined with a spectrophotometer and provides a measure for the speed of the ongoing aggregation processes [48–49]. Moreover, the turbidity, i.e., the absorbance is influenced by the size and number of aggregates formed in solution. We chose 90 mM silicic acid concentration for measurements at a reasonable timescale (up to 800 min) following Robinson et al. [48]. The silica polycondensation is very fast at the PAH concentrations applied in the experiments as shown in Figure 2 (0.45 mM) in the relevant pH range between 5 and 7. Hence, we have decreased the PAH concentration down to 31.25 µM in order to prevent rapid silica precipitation at the timescale of the turbidity measurements. The results are shown in Figure 3.

**Figure 4:**
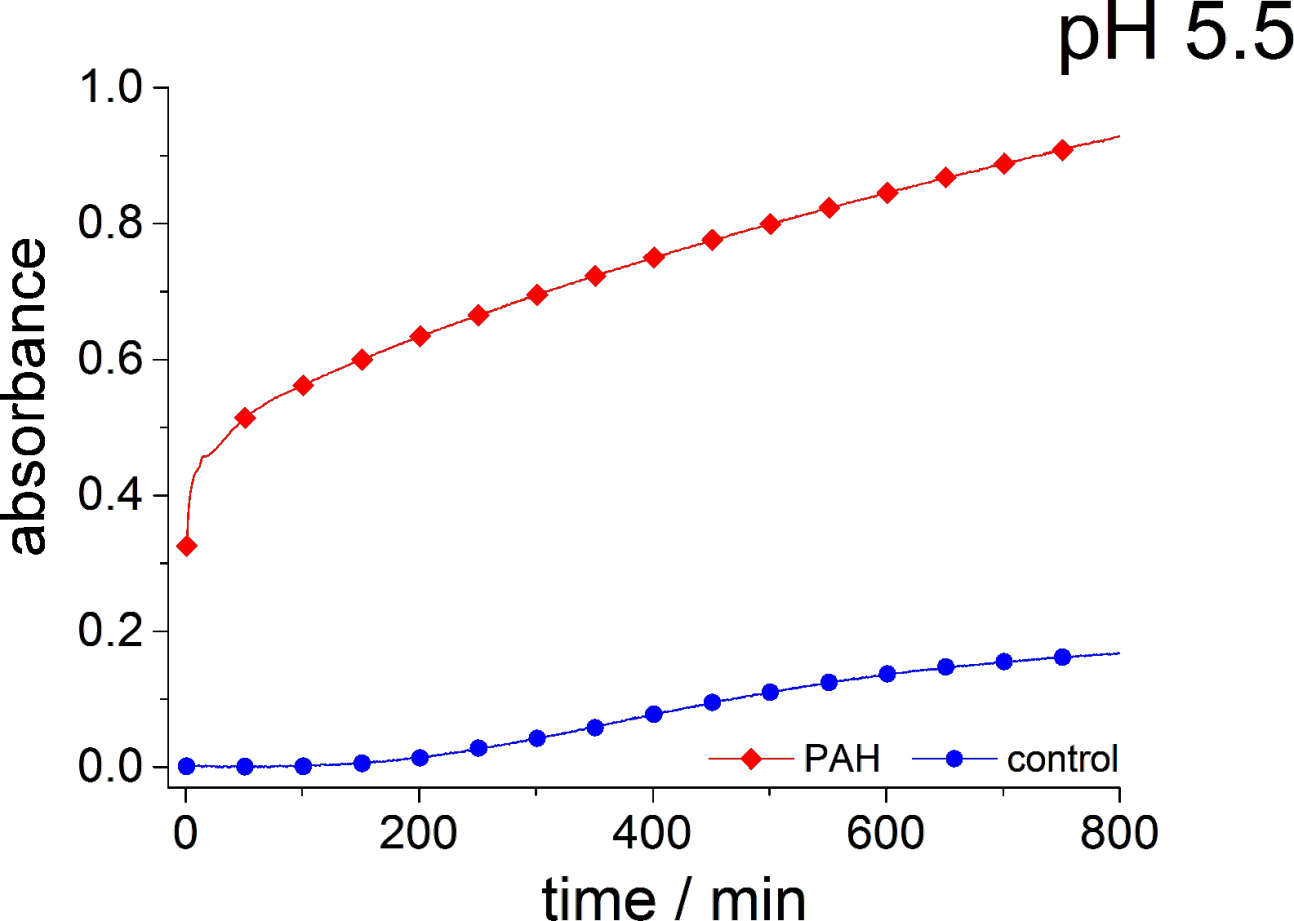
Absorbance of a 90 mM sodium metasilicate solution with 31.25 µM PAH and 180 mM hydrogen phosphate (red rhombuses) and of a sodium metasilicate solution 90 mM containing 180 mM hydrogen phosphate (control, blue circles) measured as a function of time up to 800 min.

Figure 3 displays the absorbance of a solution with pure sodium metasilicate solution (90 mM silicic acid) and a solution with 90 mM silicic acid plus 31.25 µM PAH at pH values of 5.5 and 6.8. The absorbance of the pure sodium metasilicate solution increases much faster at pH 6.8 than at pH 5.5. This is obviously due to the fact that the maximum speed of silica polycondensation is expected around pH 7. The addition of PAH to the sodium metasilicate solutions strongly enhances the absorbance at pH 6.8. That means the polyamine additive pronouncedly accelerates the aggregation process, which can be explained by the electrostatic interactions between the positively charged PAH and negative surface charges of higher silica oligomers/silica nanoparticles rapidly forming at pH 6.8. At pH 5.5, the absorbance for both samples slowly increases after an induction period of ca. 100 min. In contrast to the behavior found at pH 6.8, the addition of PAH has almost no effect at pH 5.5, the absorption of the PAH-containing sample is even slightly smaller than in the control solution. It is remarkable that this rather small change of pH by 1.3 units gives rise to such a pronounced change in the aggregation behavior. The two samples exhibit an identical overall composition except for the pH and amount of chloride resulting from the titration with HCl. With respect to the charges of the aggregating molecules, the change in pH will result in the following: The total charge of PAH may become slightly more positive. For monomeric allylamine, the p*K*_a_ value is known to be 9.49. That means allylamine would be positively charged at both pH values, 5.5. and 6.8. However, the p*K*_a_ values of polyallylamine, i.e., of allylamine in its polymeric form, are likely to be different from the monomer. Kobayashi et al. and Rao et. al. estimated a p*K*_a_ value of 9.7 for polyallylamine [44–45]. The real charge state of PAH is yet hardly predictable, but it should be supposed that a decreasing pH results in an increasingly positive charge. Moreover, the silica oligomers/nanoparticles in solution are supposed to exhibit a decreasingly negative surface charge at decreasing pH. If so, the repulsion among the increasingly positive PAH molecules would not be compensated by attractive forces with the negatively charged silica oligomers/nanoparticles. Consequently, the aggregation process would be suppressed below a certain pH, as is indeed observed for pH 5.5. If the lack of negative charges is indeed the problem, the capability of the system to self-aggregate should be restored by the introduction of negatively charged ions that themselves do not destructively interfere with the PAH/silica system. This should be the case for phosphate, which is already known to enhance silica precipitation from polyamine/silicic acid solutions. The influence of phosphate upon the sample at pH 5.5 is demonstrated in Figure 4. As predicted, the phosphate-containing solution exhibits a rapidly increasing, strong absorbance which indicates aggregate formation. In the case of the PAH-free control sample, the negatively charged phosphate has the opposite effect: The aggregation becomes even slower than in the phosphate-free sodium metasilicate solution (cf. Figure 3). This can be explained by the fact that the repulsion among the silica oligomers/nanoparticles with their negative surface charge and the phosphate ions further retards the aggregation processes. It can, therefore, be concluded that charge balance is one major parameter determining the speed of aggregation in the polyamine–silica system. Perturbed charge balance can be restored at decreasing pH by introducing phosphate or other appropriate anions into the solutions.

**Figure 5:**
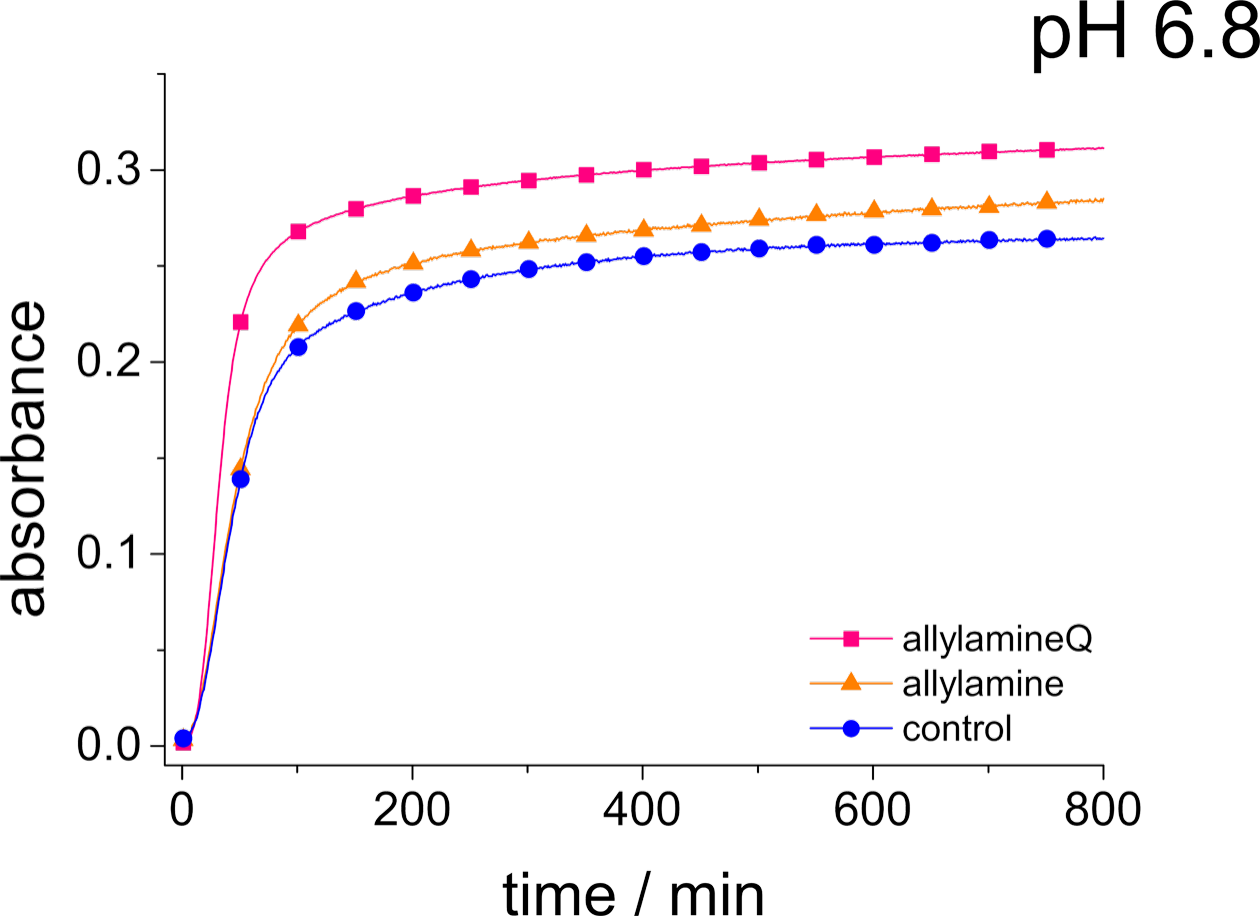
Absorbance of a 90 mM sodium metasilicate solution with 10 mM allylamine (orange triangles), 10 mM allylamineQ (pink squares) and of a sodium metasilicate solution (control, blue circles) measured as a function of time up to 800 minutes.

We have also studied sodium metasilicate solutions containing monomeric allylamine (p*K*_a_ ≈ 9.5) at the same Si/N ratio as in the PAH-containing solutions shown in Figure 3 and Figure 4 in order to elucidate possible differences between the polymeric and monomeric compounds. Moreover, monomeric allylamineQ was also used in order to analyse the influence of a quaternary ammonium group with its pH-independent, permanently positive charge surrounded by three hydrophobic methyl groups. The result of the corresponding turbidity measurements at pH 6.8 is shown in Figure 5. First of all, it is evident that the monomeric compounds are much less efficient than the polymer, PAH, in inducing the aggregation process at pH 6.8. This observation agrees with previous studies performed by Behrens et al. [50] on other polyamines. It should be noted that allylamineQ is slightly more efficient than allylamine at pH 6.8 although the charge state of both molecules should be the same (+1 elementary charges).

**Figure 6:**
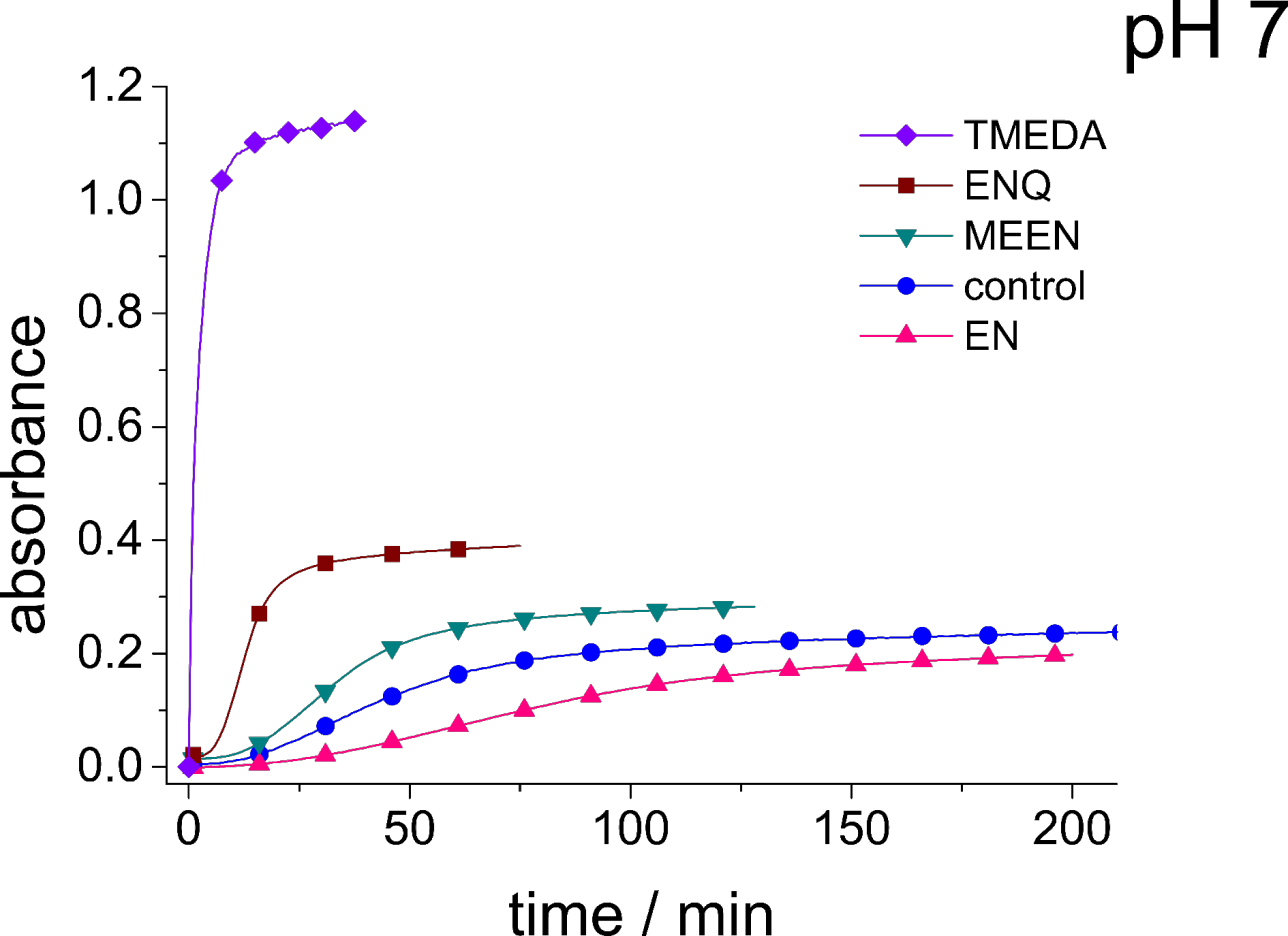
Absorbance of a 90 mM sodium metasilicate solution with 10 mM TMEDA, ENQ, MEEN, and EN (cf. Table 1) and of a pure sodium metasilicate solution (control, blue circles) measured as a function of time up to 800 min.

An explanation for the higher turbidity induced by allylamineQ compared with allylamine could be the influence of hydrophobic interactions induced by the methyl groups. This effect has already been described by Robinson et al. [48] when studying the turbidity of polyamines with different degree of methylation in solution. Interestingly, long-chain polyamines in diatoms are sometimes methylated and lysine residues in silaffins occur as trimethyllysine. It is, therefore, likely that methylation of amine moieties is an important parameter for self-assembly processes. In order to further substantiate this effect, a series of diamines with different degree of methylation was studied, the compounds and their charge state are described in Table 1. The absorbance of sodium metasilicate solutions containing these additives are displayed in Figure 6. The solution containing the compound TMEDA with two methyl groups on each of the two nitrogen atoms exhibits by far the largest turbidity. In contrast, the non-methylated compound EN exhibits the lowest absorption, even slightly below the control. The absorbance curves for two partly methylated substances MEEN and ENQ are found in between.

**Table 1:** Diamines used in the turbidity measurements and their calculated fractions of the charge states at pH 7.

pH 7	fraction of charge states [%]		

EN	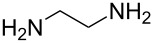	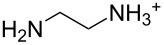	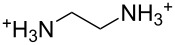
	0.04	50.56	49.40

MEEN	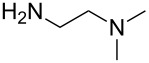	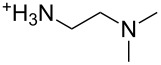	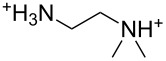
	0.21	69.95	29.84

ENQ	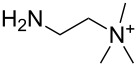	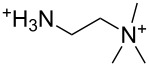	—
	40.34	59.66	

TMEDA	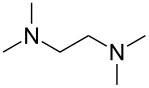	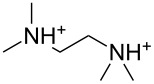	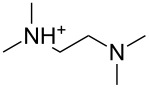
	0.75	94.51	4.74

It can, therefore, be stated that hydrophobic interactions can very strongly influence self-assembly processes taking place in silicic acid solutions. It is interesting to note in this context that Belton et al. [25] observed an increasing third order reaction rate for the monosilicic acid condensation reaction in methylated triamines compared with the non-methylated substance. It is possible, that this enhanced reaction rate is coupled with the enhanced efficiency of the self-assembly processes observed here. For polyallylamine, efficient aggregation under the chosen conditions and at pH 5.5 only occurred in the presence of phosphate (Figure 4). The final question to be answered is therefore related to the influence of phosphate upon the monomer-containing solutions.

**Figure 7:**
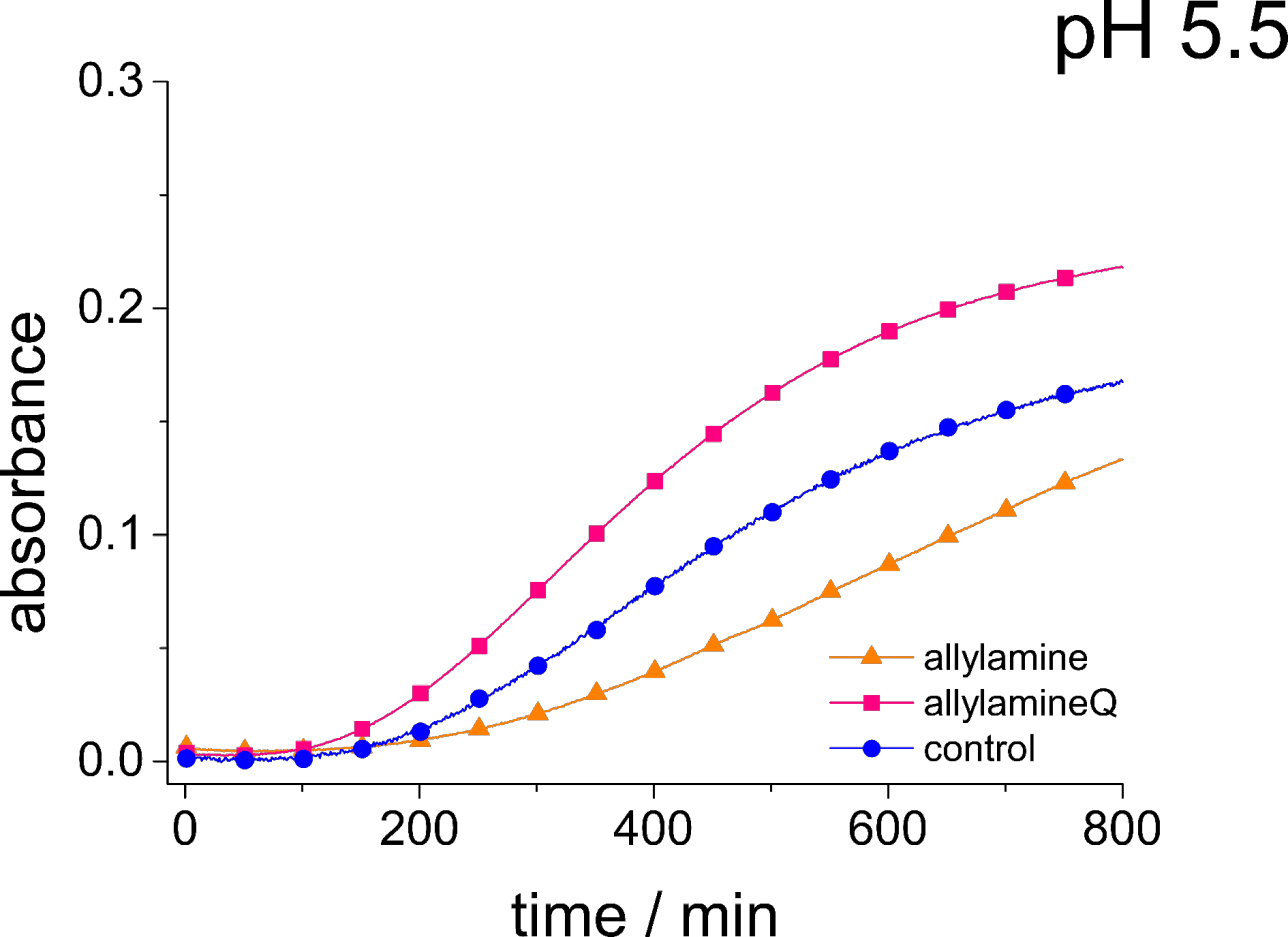
Absorbance of a 90 mM sodium metasilicate solution with 180 mM hydrogen phosphate in the presence of 10 mM allylamine and allylamineQ and of a pure sodium metasilicate solution with 180 mM hydrogen phosphate (control, blue circles) measured as a function of time up to 800 min.

Figure 7 shows the absorbance curves for phosphate-containing sodium metasilicate solutions in the presence allylamine and allylamineQ as well as for the pure phosphate-containing sodium metasilicate (control). In contrast to the behavior observed for PAH, phosphate does not enhance the aggregate formation in these solutions. The influence of phosphate is even slightly retarding the aggregation. This observation again emphasizes the need to use polymeric additives such as PAH in order to obtain an enhanced aggregation in silicic acid containing solutions.

## Conclusion

The self-assembly processes taking place in sodium metasilicate solutions containing polyamines as well as monomeric amine compounds were studied in the presence and absence of phosphate ions. The present study was especially devoted to the characterization of the initial aggregation steps taking place in such solutions. For this purpose, turbidity measurements were employed as a simple method to detect self-assembly before silica precipitation starts. The following conclusions can be drawn from our studies:

(i) Polyallylamine (PAH) is by far more efficient in inducing self-assembly processes in silicic-acid containing solutions than equivalent amounts of its monomer, allylamine. That means efficient self-assembly necessarily requires the polymeric state of the amine as already pointed out by Behrens et al. [50]. PAH strongly reduces the concentration of soluble silica especially at elevated pH above 8.5. It is tempting to speculate that PAH catalyzes the silicic acid polycondensation reaction as suggested by Kröger et al. [1] – in particular at elevated pH. Belton et al. [25] indeed observed an enhanced reaction rate for the silicic acid polycondensation reaction in the presence of different amines at pH 6.8 whereas Behrens et al. [50] did not observe such an effect at pH 5.5. Elucidation of this possible catalytic effect and its dependence on the experimental parameters should be subject of future research.

(ii) Efficient self-assembly takes place in the pure PAH/silicic acid solution at pH 6.8. This can be explained by the electrostatic interactions between positively charged polyamines and negatively charged silicic acid oligomers/silica nanoparticles. However, changes of the pH strongly influence these processes. At pH 5.5, self-assembly in the pure silicic acid/PAH solutions is totally suppressed at the concentrations chosen here. This is explained by the perturbed charge balance caused by the decreasing negative surface charge of the silica nanoparticles at lower pH. However, the introduction of negatively charged phosphate restores the ability of the system to self-assemble. This highlights the necessity of a proper charge balance in the formed aggregates.

(iii) Methylation of the amine groups strongly enhances the tendency for self-assembly in amine-containing silicic acid solutions (see also [48]). The enhanced reactivity of methylated polyamines in the silicic acid polycondensation reaction observed by Belton et al. [25] may be related to this fact. That means the degree of methylation provides a further important “tuning” parameter for bioinspired silica synthesis approaches based on the use of LCPAs which should be further exploited in future in vitro studies.

## Experimental

### Reagents and chemicals

Polyallylamine hydrochloride (PAH; (C_3_H_8_ClN)*_n_*; *M* = 15000 g/mol, *n* ≈ 160), allylamine (C_3_H_7_N; *M* = 57.09 g/mol), ethylenediamine dihydrochloride (EN; C_2_H_8_N_2_·2HCl; *M* = 133.02 g/mol), *N*,*N*-dimethylethylenediamine (MEEN; C_4_H_12_N_2_; *M* = 88.15 g/mol), (2-aminoethyl)trimethylammonium chloride hydrochloride (ENQ; C_5_H_15_N_2_Cl·HCl; *M* = 175.10 g/mol), *N*,*N*,*N*′,*N*′-tetramethylethylenediamine (TMEDA; C_6_H_16_N_2_; *M* = 116.20 g/mol), sodium metasilicate (Na_2_SiO_3_·9H_2_O; *M* = 284.2 g/mol), as well as the reagents used for the silicomolybdic acid test (ammonium molybdate ((NH_4_)_6_Mo_7_O_24_·4H_2_O), oxalic acid (C_2_H_2_O_4_·2H_2_O)) were purchased from Sigma-Aldrich (Germany). The allyltrimethylammonium bromide (allylamineQ; C_6_H_14_BrN; *M* = 180.09 g/mol) was obtained from ABCR (Germany).

The samples studied in this work were prepared by using purified distilled water (filtersystem: Elga – Purelab Classic, Germany; filter: Gelman Sciences – Supor^®^ DCF^TM^ 0.2 µm). In the following, this deionized water will be called ultrapure water.

### Silicomolybdic acid test

The solutions for the silicomolybdic acid test were prepared and used by following the protocol developed by Spinde et al. [23].

#### ^29^Si NMR measurements

To obtain ^29^Si-enriched sodium metasilicate (Na_2_^29^SiO_3_), ^29^SiO_2_ was melted with sodium carbonate (Fluka), thus forming Na_2_^29^SiO_3_ in a solid-state reaction.

For liquid-state ^29^Si NMR measurements, 24.2 mg of Na_2_^29^SiO_3_ were dissolved in 2 mL of D_2_O/H_2_O (1:1) and placed in a container with a Teflon-covered magnetic stirring bar, resulting in a 6030 ppm SiO_2_ stock solution at pH 12. For the acidic sample, 24.2 mg of Na_2_^29^SiO_3_ were dissolved in 2 mL 0.25 M hydrochloric acid and hydrolyzed for 15 min. Ultrapure water was added to both stock solutions giving a final silicic acid concentration of 4350 ppm.

^29^Si NMR experiments were performed on a Bruker Avance 300 spectrometer operating at a resonance frequency of 59.63 MHz. For liquid-state ^29^Si NMR measurements, a commercial 10 mm HR probe (56° flip angle, number of scans 180, 60 s repetition time) was used. Typical *T*_1_ values for samples in solution were 8–13 s. Waltz16 ^1^H decoupling was applied during signal acquisition. The chemical shift was referenced relative to tetramethylsilane (TMS).

#### pH Titration

The starting solutions were prepared by mixing 2.1 mL of orthosilicic acid solution (100 mM, Na_2_SiO_3_) with 0.6 mL of a PAH containing solution (2 mM) or ultrapure water (control). The final ratio of silicon and nitrogen atoms in the polymer-containing sample was 1:1 and the starting pH was 12.7. The desired pH values were adjusted by titration with a 2.4 M HCl stock solution under continuous stirring. The final SiO_2_ concentration was 70 mM. The resulting solution (control) or precipitate (PAH) were transferred into Eppendorf vials and set aside without stirring. The concentration of soluble silicic acid was determined using the well-established silicomolybdic acid test 40 min after titration.

#### Turbidity measurements

To slow down the reaction, the final ratio of silicon and nitrogen atoms in the turbity measurements was changed to 9:1. Turbidity measurements were performed by mixing a silicate-containing solution A with different amines (solution B).

**Preparation of solution A without phosphate:** Solution A was prepared by titration of a stock solution of sodium metasilicate (ca. 250 mM) to pH 6.8 or 5.5 with 2.5 M hydrochloric acid (see final concentrations in Table 2). Finally, the samples were diluted to a Si-concentration of 120 mM.

**Table 2:** Final concentrations of stock solution A without phosphate.

final concentration (solution A without phosphate)	pH 5.5	pH 6.8

silica	120 mM	120 mM
chloride	220 mM	180 mM

**Preparation of solution A with phosphate:** Solution A was prepared as described by using 0.5 M phosphoric acid for titration (see final concentration in Table 3). Finally, the samples were diluted to a Si-concentration of 120 mM.

**Table 3:** Final concentrations of stock solution A with phosphate.

final concentration (solution A with phosphate)	pH 5.5	pH 6.8

silica	120 mM	120 mM
phosphate	240 mM	160 mM

**Preparation of solution B:** The amine-containing solution B was prepared by titration of an amine stock solution (ca. 40 mM or ca. 0.25 mM for PAH) with 2.5 M hydrochloric acid. Afterwards, the solutions were diluted to the final concentrations shown in Table 4.

**Table 4:** Final concentrations of stock solution B.

final concentration (solution B)		pH 5.5	pH 6.8

amine	monomer	20 mM	20 mM
	polymer	125 µM	125 µM
HCl		depending on amine	

**Measurements:** All solutions were prepared immediately before use. Both solutions were stored in an ice bath to slow down further reactions. Before starting the measurements, the samples were warmed in a water bath at room temperature for 5 min. Mixing of 1.2 mL solution A with 0.4 mL of solution B resulted in the final concentrations displayed in Table 5 and Table 6.

**Table 5:** Final concentration of turbidity measurement samples without phosphate.

final concentration (without phosphate)		pH 5.5	pH 6.8

silica		90 mM	90 mM
amine	monomer	10 mM	10 mM
	polymer	31.25 µM	31.25 µM
chloride		165 mM	135 mM

**Table 6:** Final concentration of turbidity measurements with phosphate.

final concentration (with phosphate)		pH 5.5	pH 6.8

silica		90 mM	90 mM
amine	monomer	10 mM	10 mM
	polymer	31.25 µM	31.25 µM
phosphate		180 mM	120 mM

An initial absorption spectrum was taken from 400 to 500 nm on a Varian Cary 50 spectrometer. The solutions were directly mixed in a glass cuvette, shortly shaken and the measurement started immediately. For rapidly reacting solutions (such as with PAH) solution B was given directly into the cuvette, which already contained solution A and was placed in the spectrometer by moving the pipette from the bottom upwards. The resulting mixture was homogenous and no air bubbles, gradient or sedimentation could be observed. The absorbance was measured as a function of time (*t*_max_ = 800 min) in continuous mode every minute. Measurements were run overnight. The absorbance at 480 nm was taken as a measure of turbidity.
